# Evaluation of knowledge of healthcare workers in hospitals of Zabol city on proper methods of blood and components transfusion

**DOI:** 10.4103/0973-6247.53878

**Published:** 2009-07

**Authors:** Piri Ali Reza, Shahraki Vahed Aziz, Moien Abbas Ali, Mardani Hamuleh Marjan, Taghavi Mohammad Reza

**Affiliations:** *Department of Nursing & Midwifery, Zabol Medical Science University, Islamic Republic of Iran (I.R.Iran)*

**Keywords:** Blood transfusion, healthcare worker, knowledge

## Abstract

**Background and Aims:**

Blood and components are more frequently used in surgery and non-surgical procedures. In medical procedures blood transfusion is important and needs adequate expertise and practice, thus adequate knowledge in healthcare workers of this procedure is essential.

**Materials and Methods:**

This descriptive study is designed to assess the knowledge of healthcare workers about proper methods of blood transfusion, and how to promote their knowledge for proper performance if their knowledge is inadequate. Data were collected with aimed questionnaire and analyzed by statistics software.

**Result:**

The study population mainly comprised 122 healthcare workers (HCWs). The main findings from this study showed that 26.2% of healthcare workers (HCWs) had low-level knowledge, 22.1% moderate and 51.6% acceptable knowledge. We did not find any significant correlation between knowledge of HCWs and years in profession, participation in training, number of blood transfusions per day, age, gender, etc. (*P* < 0.05).

**Discussion and Conclusion:**

Results strongly emphasized the need for a curriculum to promote knowledge of HCWs about blood transfusion because we found low and moderate level of knowledge in approximately half our samples and on the importance of blood transfusion procedure, suggesting that more attempts should be made to build up knowledge about blood transfusion.

## Introduction

The practice of blood transfusion is the transference of blood from the circulation of one individual to another for practical therapeutic purposes.[1] Blood transfusion was performed with excessive complications in the beginning of the 20th century. Today these complications are limited by increase in the knowledge of HCWs and knowledge of blood function.[2] Error in transfusion is one of the original causes of mortality thus awareness of this error is important.[3] Nowadays blood and components are used more frequently in surgery and non-surgical procedures. In medical procedures blood transfusion is important and needs adequate skills. Adequate blood and components provision is of problematic status in medicine, however, today this problem has been resolved by promoting blood and components screening programs.[4] Nevertheless some of these problems remain.[5] Security, safety and trust in transfusion are variable.[6] Guidance about blood and components depends on the expertise of personnel.[7] Blood transfusion is an elaborate procedure and performing this procedure for a patient requires adequate skills and knowledge.[8] HCWs and other personnel who work with them are two of the original safe performers in transfusion procedures.[9] Ingrand *et al*. in their experimental study on a stratified sample of 48 nurses, showed that erroneous decisions oc-curred in 18.2% of the 576 blood compatibility tests per-formed at the bedside and showed this underscored the importance of continuing efforts to update theoretical and practical knowledge of nurses about this transfusion safety procedure.[10] Another study describing knowledge and practice of nursing staff in four French hospitals reported consistent deficiencies in knowledge and practice about labeling of tubes during phlebotomy for immunohematological testing, in blood product conservation on the ward, and in application of the bedside blood com-patibility test.[11] In another descriptive study on blood transfusion knowledge and practice among nurses, Bayraktar and Erdil showed insufficient knowledge about blood transfusion was reflected in undesirable practice.[12] In addition, in another study in Canada by Hardy on current status of transfusion triggers for red blood cell concentrates showed future research and development should focus on the determination of optimal transfusion strategies in various patient populations and on reliable monitors to guide transfusion therapy.[13] Thus studies have proved that adequate knowledge in HCWs on this procedure is essential. Hence this descriptive study performed to evaluate the knowledge of HCWs in hospitals of Zabol city in the Islamic Republic of Iran on proper methods for blood and components transfusion.

## Materials and Methods

For this descriptive study, the population consisted of 122 HCWs (nurses, midwives, operation technicians and other staff) working in the hospitals of Zabol city in the Islamic Republic of Iran. We drew a random sample using a proportional allocation (proportional to the total number of HCWs in each hospital), and stratified by the type of ward (medicine, surgery, emergency, operation room, maternity, polyvalent). Polyvalent HCWs are HCWs not attached to a single ward; they rotate one ward to another, depending on need. When HCWs could not or refused to participate, other HCWs were drawn from the same stratum. Data were collected anonymously at the hospitals by a trained investigator who led structured individual interviews with the HCWs. Appointments with HCWs were made through the hospital supervisor and not directly with the interviewed HCWs who did not know in advance either the theme or the date of the interview. To decrease the risk of information diffusion, the investigator recommended that each HCW interviewed talk about neither the theme nor the content of the questionnaire before the end of data collection; data collection never lasted more than five consecutive days in a given hospital. The questionnaire contained 38 closed questions included demographic questions (9 questions) and knowledge survey questions (29 questions). These questions were approved by a Medical Sciences faculty member of Zabol University. Knowledge questions in the questionnaire included four parts: 1- Knowledge of procedure before transfusion 2- Knowledge of essential medical tests for blood and components transfusion 3- Knowledge of procedure during transfusion 4- Knowledge of complications of blood and components transfusion. Demographic data included age, gender, years in profession; participation in training etc. We scored the answers of the questionnaire on three levels, good, moderate and weak by manual computation and then converted to digital form to compute by computer.

### Statistical analysis

Descriptive analysis consisted of frequencies and % iles or means and standard deviations, depending on the level of the data. Chi-square tests, two-sample t-tests and correlations were used to assess associations between selected sociodemographic factors, including age, gender, years in profession, participation in training etc. versus differences in knowledge of blood and components transfusion. These tests were conducted using a level of statistical significance of 0.05.[14,15] All analyses were conducted using SPSS V.13.0 (SPSS, Inc. Chicago, IL).

## Results

**Figure 1:**
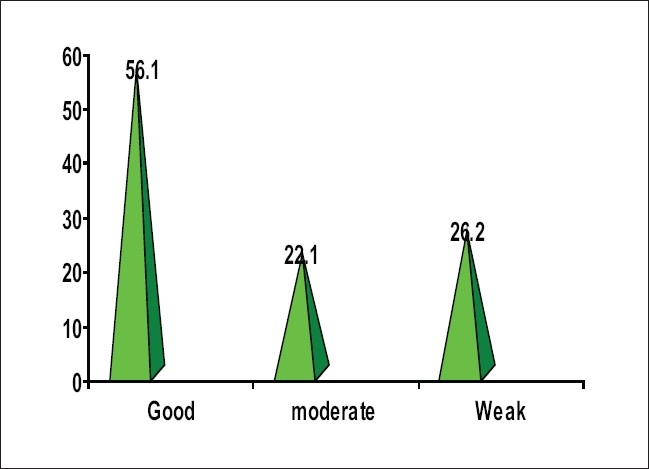
Frequency of knowledge of HCWs of correct method of blood and components transfusion

In total 122 HCWs (48 male and 74 female) who worked in hospitals of Zabol city were interviewed by the investigator. Ninety-two of them were nurses, three midwives, 10 operation technicians and 17 other staff (these personnel work in hospitals and participated in treatment procedure). Their years in profession (the years worked after being hired in hospital) were in the range of three months to 30 years. 12.2 percent of them have 5 years experience in the profession. According to a numbering scale the knowledge level was proportioned in three parts (good, moderate and weak); in total 26.2% had weak level knowledge, 22.2% moderate knowledge and 51.6% good knowledge. Responses to questions about complications of blood and component transfusion were 35.2% good, 22.1% moderate, and 19% weak. Responses to questions on procedure before transfusions were 59% good, 22.1% moderate and 38.6% weak. Responses to questions procedure during transfusion were 68.8% good, 14.8% moderate and 16.8% weak. Responses to questions on essential medical tests for blood and components transfusion were 22.1% good, 23% moderate and 54.9% weak [Table 1]. Only 24.6% of HCWs who underwent training have good knowledge on blood transfusion [Table 2]. Twenty-three percent of good responses on complications of blood and components transfusion by female responders have significant correlation in a sample T test (*P* < 0.5) [Table 3]. We did not find any another significant correlation between knowledge of HCWs of each part of the questionnaire and demographic data by either statistical test [Table 4] [Figure 1].

**Table 1:** Distribution of knowledge in three levels

Knowledge score kind of knowledge	Good		Moderate		Weak		Total	
	N Percent	N Percent	N Percent	N Percent				
General knowledge about proper method of blood transfusion	63	51.6	27	22.2	32	26.2	122	100
Knowledge about complications of blood and component transfusion	43	35.2	32	26.2	47	38.6	122	100
Knowledge about procedure before transfusion	72	59	27	22.1	23	18.9	122	100
Knowledge about procedure during transfusion	84	68.8	18	14.8	20	16.4	122	100
Knowledge about essential medical tests for blood and component transfusion	27	22.1	28	23	67	54.9	122	100

**Table 2:** Frequency table between knowledge of HCWs and participation in training*

Participation in training knowledge score	Participated in training		Did not participate in training	

	N	Percent	N	Percent
Good	30	24.6	33	27.1
Moderate	10	8.2	17	14
Weak	7	5.8	25	20.5

* This training provided for promoting the knowledge of HCWs of blood and components transfusion at the University of Zabol in December 2005)

**Table 3:** Frequency table between knowledge of HCWs toward complication of blood and components transfusion and gender

Gender knowledge score	Male		Female	

	N	Percent	N	Percent
Good	15	12.3	28	23
Moderate	19	15.5	13	10.6
Weak	14	11.5	33	27.1

*Correlation was significant with *P* < 0.05 by sample T test

**Table 4:** Frequency table between years in profession and knowledge

Years in profession knowledge score	3 (months) to 10 (years)		10 to 20 (years)		20 to 30 (years)	
	N	Percent	N	Percent	N	Percent
Good	41	33.6	18	14.8	4	3.3
Moderate	18	14.8	4	3.3	5	4.1
Weak	19	17.6	7	7.5	6	5

No correlation found

## Discussion

This study describes knowledge pat-terns related to proper method of blood transfusion among HCWs who participated in blood transfusion, and identified poor knowledge that could be direct threats to patients. These threats concerned poor knowledge of procedure before transfusion, essential medical tests for blood and components transfusion, procedure during transfusion and complications of blood and components transfusion. Our results in this study showed that approximately half of HCWs have weak and moderate knowledge of proper methods of blood and components transfusion. A training curriculum must be proposed in the hospitals of Zabol for improving knowledge of HCWs about blood and component transfusion. Some authors, however, have suggested that continuing professional education cannot improve performance, which is confirmed by our finding which showed no correlation between knowledge of HCWs and years in profession, or participation in training.[16,17] Studies have shown the effectiveness of educational outreach visits or of repeated continuing professional education in improving the appropriateness of blood product prescription by physicians.[18‐19] Factors related to the effectiveness of continuing professional education in changing nurses’ practice patterns are linked to: (1) participants (their motivation or willingness to change); and (2) the type of training (targeted at nurses’ needs, integrated into the work environment)[20‐24] Similar factors have been found in studies of the effectiveness of continuing medical professional education[25,26] As regards our results we recommend more than mere strategy to improve the knowledge of HCWs particularly for nurses. Thus we recommend:

1. All nurses receive training in blood transfusion;
2. Only nurses who have been trained and have specific qualifications in blood transfusion medicine are allowed to practice it;
3. Nurse training curricula reflect the requirements of modern transfusion medicine and other specialized fields of medicine such as oncological and hematological disorders, surgical procedures, autologous donation of blood, as well as bone marrow and organ transplantation;
4. Implementation and evaluation of continuous training programs is carried out in order to improve the quality and safety of blood transfusion;
5. Mechanisms are developed for cooperation between nurses, physicians, and other healthcare workers employed in hospitals, blood establishments, and hospital blood banks;
6. Procedures are set up to monitor knowledge of key processes, such as clinical audit, with ongoing feedback and implementation of remedial action, to ensure continuous improvement in performance;
7. Guidelines and procedural protocols in blood transfusion medicine for the nursing staff and other professionals are developed in accordance with relevant Council of Europe recommendations.
